# Development of a toolbox to dissect host-endosymbiont interactions and protein trafficking in the trypanosomatid *Angomonas deanei*

**DOI:** 10.1186/s12862-016-0820-z

**Published:** 2016-11-11

**Authors:** Jorge Morales, Sofia Kokkori, Diana Weidauer, Jarrod Chapman, Eugene Goltsman, Daniel Rokhsar, Arthur R. Grossman, Eva C. M. Nowack

**Affiliations:** 1 Department of Biology, Heinrich-Heine-Universität Düsseldorf, Universitätsstr. 1, 40225 Düsseldorf, Germany; 2 Plant Genome Group, DOE Joint Genome Institute, 2800 Mitchell Drive, 94598 Walnut Creek, CA USA; 3 Department of Plant Biology, Carnegie Institution for Science, 260 Panama Street, 94305 Stanford, CA USA

**Keywords:** Endosymbiosis, Bacterial endosymbiont, Protein targeting, Homologous recombination, Protist, Trypanosomatid

## Abstract

**Background:**

Bacterial endosymbionts are found across the eukaryotic kingdom and profoundly impacted eukaryote evolution. In many endosymbiotic associations with vertically inherited symbionts, highly complementary metabolic functions encoded by host and endosymbiont genomes indicate integration of metabolic processes between the partner organisms. While endosymbionts were initially expected to exchange only metabolites with their hosts, recent evidence has demonstrated that also host-encoded proteins can be targeted to the bacterial symbionts in various endosymbiotic systems. These proteins seem to participate in regulating symbiont growth and physiology. However, mechanisms required for protein targeting and the specific endosymbiont targets of these trafficked proteins are currently unexplored owing to a lack of molecular tools that enable functional studies of endosymbiotic systems.

**Results:**

Here we show that the trypanosomatid *Angomonas deanei*, which harbors a β-proteobacterial endosymbiont, is readily amenable to genetic manipulation. Its rapid growth, availability of full genome and transcriptome sequences, ease of transfection, and high frequency of homologous recombination have allowed us to stably integrate transgenes into the *A. deanei* nuclear genome, efficiently generate null mutants, and elucidate protein localization by heterologous expression of a fluorescent protein fused to various putative targeting signals. Combining these novel tools with proteomic analysis was key for demonstrating the routing of a host-encoded protein to the endosymbiont, suggesting the existence of a specific endosymbiont-sorting machinery in *A. deanei*.

**Conclusions:**

After previous reports from plants, insects, and a cercozoan amoeba we found here that also in *A. deanei*, i.e. a member of a fourth eukaryotic supergroup, host-encoded proteins can be routed to the bacterial endosymbiont. This finding adds further evidence to our view that the targeting of host proteins is a general strategy of eukaryotes to gain control over and interact with a bacterial endosymbiont. The molecular resources reported here establish *A. deanei* as a time and cost efficient reference system that allows for a rigorous dissection of host-symbiont interactions that have been, and are still being shaped over evolutionary time. We expect this system to greatly enhance our understanding of the biology of endosymbiosis.

**Electronic supplementary material:**

The online version of this article (doi:10.1186/s12862-016-0820-z) contains supplementary material, which is available to authorized users.

## Background

The impact of bacterial endosymbionts on eukaryote evolution and the Earth’s biogeochemical cycles is highlighted by the ubiquitous occurrence of mitochondria and plastids, organelles that evolved as a consequence of endosymbiosis more than a billion years ago [1, 2]. In the last two decades it has become apparent that there are more recently established bacterial endosymbionts found across the eukaryotic tree of life [3, 4]. These associations engage a broad range of bacterial phyla, represent various levels of integration, and usually provide the host with new physiological capabilities that allow it to colonize and propagate in ecological niches that would otherwise be inaccessible [5].

Advances in genome sequencing technologies have been exploited to greatly enhance our understanding of the physiological basis of many endosymbiotic interactions. The genomes of bacterial endosymbionts tend to become reduced, sometimes down to organellar genome sizes, while functions that are beneficial for the host are retained [6, 7]. Furthermore, the gene repertoire of the host and endosymbiont can become highly complementary, which indicates integration of metabolic processes between the partner organisms [8–10]. Molecular mechanisms that mediate these interactions, however, are only starting to be unveiled. While endosymbionts where initially expected to exchange only metabolites with their host cells, recent evidence has demonstrated that it is not only metabolites, but also host-encoded proteins that can be targeted from the cytoplasm of the host cell to the bacterial symbiont. In the cercozoan amoeba *Paulinella chromatophora* >30 genes originally derived from its cyanobacterial endosymbiont (now an organelle called a chromatophore) were identified in the host nuclear genome [11]. Transcripts from three of these genes were shown to be translated on cytoplasmic ribosomes and their protein products targeted to the chromatophore where they assembled with the chromatophore-encoded subunits of photosystem I [12]. In certain plants and insects, nuclear-encoded peptides have been shown to be routed into their endosymbionts where they regulate/modulate endosymbiont growth and division [13–15].

However, understanding the molecular mechanisms that underlie metabolic complementation and coordination, protein targeting and import, signaling between symbiotic partners, and synchronization of host and endosymbiont cell cycles is limited. This limitation reflects the lack of sophisticated molecular/genetic tools that can be used to probe endosymbiotic associations, the intrinsic complexity of the multicellular systems that are being explored (e.g. symbiosis in insects and plants), and the need to invest time and resources for establishing and maintaining genetically-modified organisms [16, 17]. Therefore, developing a molecular toolbox for querying an endosymbiont-harboring protist that is easily grown and has a short generation time would represent a considerable asset to the field of symbiosis research.

The trypanosomatid *Angomonas deanei* belongs to the Kinetoplastea, a class that includes clinically and economically important pathogens such as *Leishmania* spp., *Trypanosoma brucei,* and *Trypanosoma cruzi. Angomonas* along with the genera *Strigomonas* and *Kentomonas* form a monophyletic clade within the Kinetoplastea, the subfamily Strigomonadinae that is characterized by the presence of a single β-proteobacterial endosymbiont in their cytoplasm [18]. The endosymbiont is enclosed by two membranes and a reduced peptidoglycan layer [19], divides synchronously with the host cell, and is vertically transmitted to progeny cells [20]. Whereas most trypanosomatids are nutritionally fastidious and have a strict requirement for heme and several amino acids, members of the Strigomonadinae can grow in defined media lacking heme and containing a reduced number of amino acids because many metabolites can be synthesized by the endosymbiont and delivered to the host [21–23]. Besides members of the Strigomonadinae, there is a single trypanosomatid species (*Novymonas esmeraldas*) that is known to have acquired bacterial endosymbionts independently [24].

The full genome of *A. deanei* and its β-proteobacterial endosymbiont were recently sequenced [22, 25]. The 0.8 Mb endosymbiont genome is strongly reduced compared to free-living β-proteobacteria and the complement of encoded proteins suggests tight metabolic cooperation between the endosymbiont and host cell [22, 25]. As is typical for trypanosomatids, the nuclear genome of *A. deanei* is characterized by a lack of introns and transcription of long polycistronic mRNAs that mature by cleavage into single open reading frames (ORFs) concomitant with the addition of a splice leader (SL) at their 5′-end and polyadenylation at their 3’-end. The relatively simple trypanosomatid genome organization, with typically high levels of homologous recombination (HR), has allowed for the development of numerous molecular biological tools to probe trypanosomatid physiology [26–28].

*A. deanei* has a doubling time of ~6 h in standard nutrient broth (see Methods), making propagation and experimentation both efficient and inexpensive. In addition, the genomic structure and availability of molecular tools for dissecting closely related organisms make *A. deanei* a promising endosymbiotic model for developing a powerful system for in depth exploration of molecular mechanisms that govern host-endosymbiont interactions. Here we describe the stable integration of transgenes into the *A. deanei* genome, high level expression of those genes, and the generation of null mutants. Furthermore, combining proteomic analysis with the newly developed molecular capabilities, we demonstrate that the host Endosymbiont-targeted Protein 1 (ETP1) is routed to the endosymbiont suggesting the existence of a sorting machinery in *A. deanei* that delivers proteins to the endosymbiont.

## Results

### Genome and transcriptome assemblies set the foundation for developing genetic tools

Since the available draft assembly of the *A. deanei* nuclear genome is still highly fragmented (contig N50 = 2.5 kb; [25]), information important for building molecular tools for this protist, including sequences of long genomic contigs spanning multiple genes and expression levels of specific genes, is missing. Therefore, a new genome assembly was generated for *A. deanei* ATCC PRA-265. In total, 408 scaffolds totaling 19.3 Mbp of sequence were assembled. These scaffolds comprised 1,884 contigs of total length 17.6 Mb, and 1.7 Mb of intra-scaffold gaps (9.0 % of the assembly). Half of the assembly is in 22 scaffolds longer than 300.8 kb (the scaffold N50 length) and 177 contigs are longer than 28.8 kb (the contig N50 length). This assembly provides more than 10-fold improvement in linkage relative to the gene-oriented assembly that was already available. This Whole Genome Shotgun project has been deposited at DDBJ/ENA/GenBank under the accession LXWQ00000000. Expression levels of many nuclear genes were inferred from RNAseq analyses (see Additional file 1: Table S1).

### Efficient homologous recombination in *A. deanei*

We hypothesized that, like for other trypanosomatids, *A. deanei* would be amenable to genetic manipulation using HR and transgene expression would occur by read through without the need for a dedicated promoter [28]. The loci for one gene of each, the γ- and δ-subfamily of amastins [transcripts “a66;8439” and “a96;12664”, respectively (see Additional file 2: Figure S1a); hereafter referred to as γ- and δ-amastin], were selected for the site at which a cassette containing a drug resistance marker gene would be inserted because (i) these two amastin genes are highly expressed (Additional file 1: Table S1), (ii) amastin loci have been previously used to enhance expression of heterologous genes in other trypanosomatids [29, 30], and (iii) a knockdown of δ-amastin does not seem to affect proliferation of insect stages in other trypanosomatid species [31]. Amastins are surface glycoproteins of unknown function that can be grouped into four subfamilies (termed α to δ) and occur in some trypanosomatids as tandemly arrayed multicopy genes [32]. Several members of each subfamily are encoded on the *A. deanei* genome [25], but sequences of the various members of the γ- and δ-subfamilies differ at the nucleotide and amino acid levels and their intergenic regions show no homology (Additional file 2: Figure S1b, c).

**Fig. 1:**
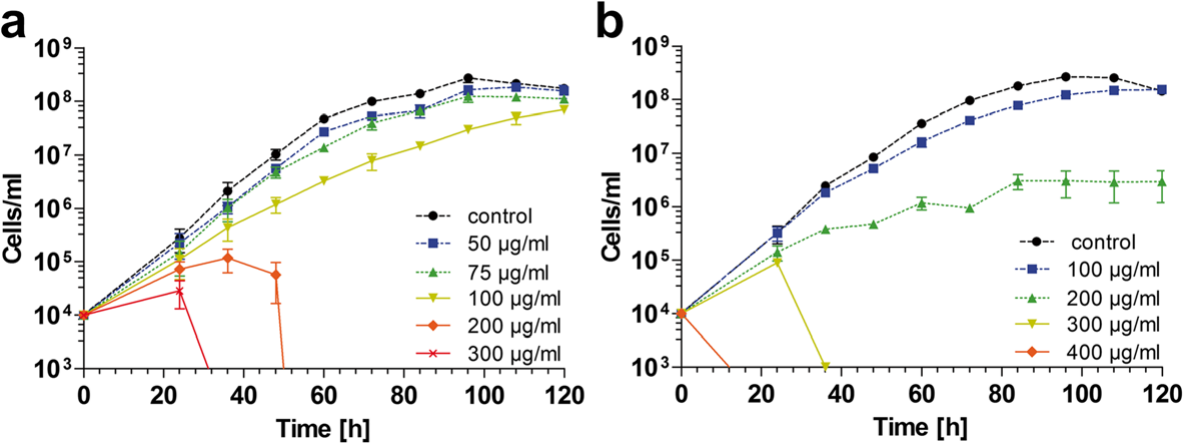
**a** G-418 and (**b**) hygromycin sensitivity of *A. deanei*. Cultures of wild-type cells grown to mid-log phase were diluted in 10 ml of fresh medium supplemented with the indicated concentration of drug to 1 × 10^4^ cell/ml and incubated at 28 °C without agitation. Cells were counted every 12 h and the average and standard deviation from three independent cultures were plotted against time

**Fig. 2:**
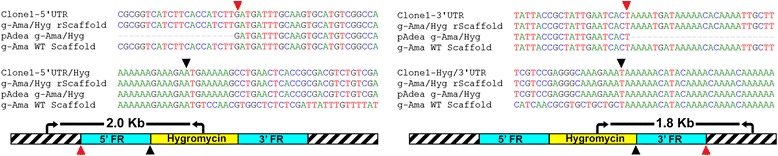
Sequence analysis confirms insertion of resistance cassette in the γ-amastin locus. Genomic DNA extracted from Δ-γ-ama^*Hyg*^ SKO clone 1 (see Additional file 2: Figure S3a) was used as a template for PCR with primers binding to the gDNA outside of the cassette and inside the resistance markers. The PCR products obtained were sequenced. The alignment shows: Clone1-5′ or 3′ UTR, sections of the genomic sequence of the transformant resulting from HR between the gDNA and the resistance cassette (pAdea γ-Ama/Hyg); clone1-5′UTR/Hyg and clone1-Hyg/3′UTR, sections of the genomic sequence of the transformant surrounding the start and stop codon of the *hyg* gene, respectively; γ-Ama/Hyg rScaffold, the expected genomic sequence organization of the recombinant Δ-γ-Ama^*Hyg*^ locus; γ-Ama WT Scaffold, wild-type sequence at the γ-amastin locus. The map underneath the alignment indicates primer binding sites (*arrows*) and sizes of resulting PCR products. Red arrowheads indicate the position at which the cassette inserted by HR into the genome and black arrowheads denote the start and stop codon of the *hyg* and γ-amastin ORFs. The striped rectangles represent genomic regions upstream and downstream of the insertion sites for the cassettes. Light blue rectangles represent the 5′- and 3′-flanking regions (FR) of the γ-amastin gene that are present in the cassette. Yellow rectangles represent *hyg*

**Fig. 3:**
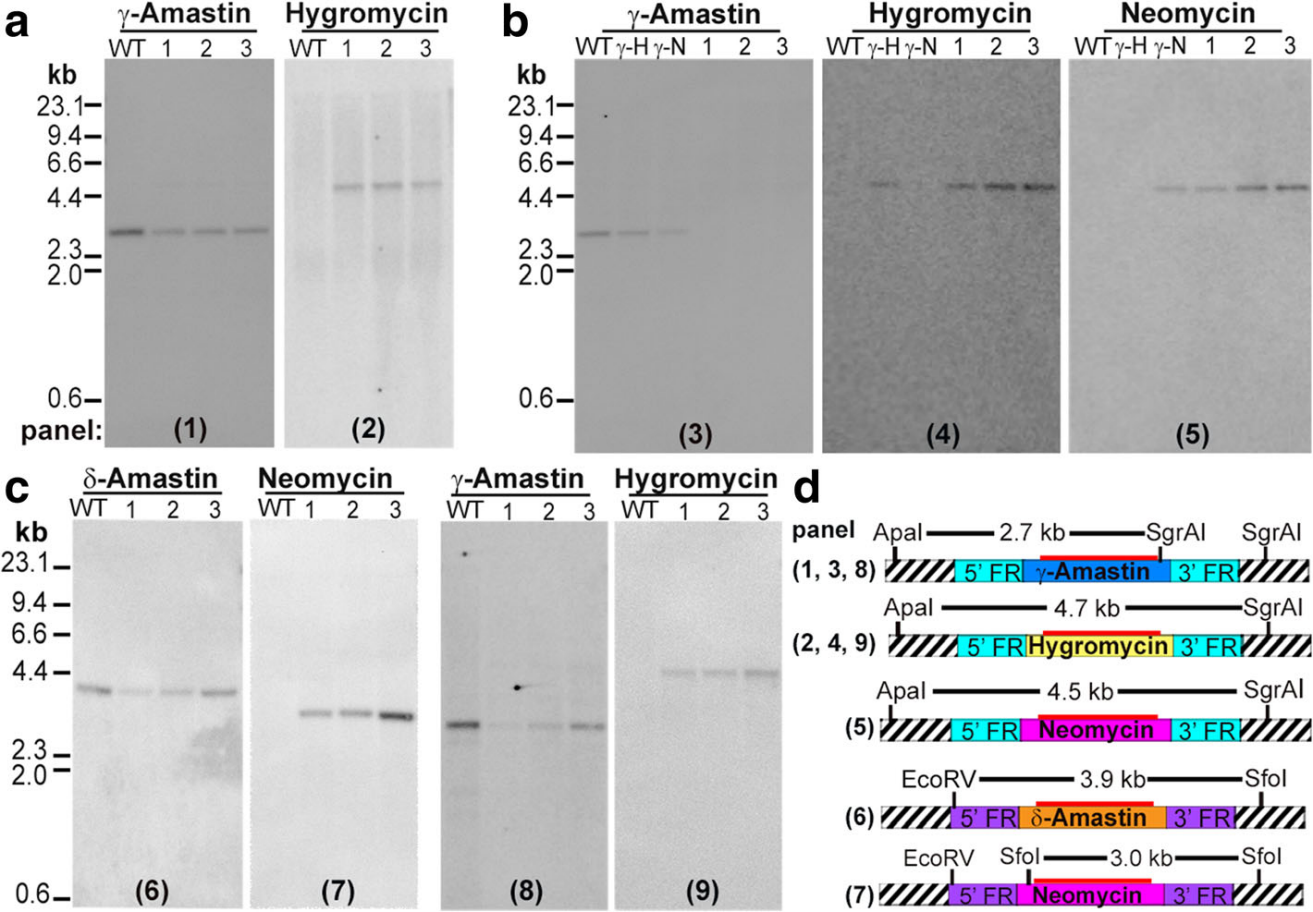
Homologous recombination in *A. deanei*. Genomic DNA was restricted with specified enzymes and analyzed by Southern blot hybridizations using probes against γ-amastin, δ-amastin, *hyg*, or *neo*, as indicated above each blot. The wild type (WT) and three clones (1, 2, and 3) were analyzed for Δ-γ-ama^*Hyg*^ SKO (**a**), Δ-γ-ama^*Hyg*^/Δ-γ-ama^*Neo*^ DKO (**b**), and Δ-γ-ama^*Hyg*^/Δ-δ-ama^*Neo*^ PKO cell lines (**c**). (**a**) In the Δ-γ-ama^*Hyg*^ SKO cell lines, a band shift of 2 kb between the γ-amastin WT locus and the recombinant locus containing *hyg* could be readily observed in all three clones analyzed, indicating replacement of the first γ-amastin allele by *hyg*; (**b**) a second transfection with a cassette encoding *neo* targeting the γ-amastin locus completely abolished the γ-amastin WT locus in all three Δ-γ-ama^*Hyg*^/Δ-γ-ama^*Neo*^ DKO clones analyzed; as a control, gDNA from the SKO lines Δ-γ-ama^*Hyg*^ (γH) and Δ-γ-ama^*Neo*^ (γN) was tested; (**c**) transfection of Δ-γ-ama^*Hyg*^ SKO clone 1 with a cassette encoding *neo* targeting the δ-amastin locus yielded the Δ-γ-ama^*Hyg*^/Δ-δ-ama^*Neo*^ PKO cell lines. (**d**) Restriction maps are provided for the various blots. The striped rectangles represent genomic regions up and downstream of the insertion sites for the cassettes, light blue and violet rectangles represent the 5′- and 3′-flanking regions (FR) of the γ- and δ-amastin genes, respectively, that are present in the cassettes; blue and orange rectangles represent the γ- and δ-amastin ORFs, respectively; and *hyg* and *neo* are represented by yellow and pink rectangles, respectively. The red bar above the inserted marker gene highlights the binding region for the DNA probe used for hybridization

**Fig. 4:**
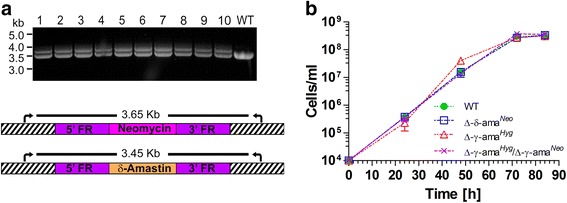
(**a**) Stability of genomic insertion in *A. deanei* and (**b**) knock-out of the amastin genes does not affect growth of *A. deanei*. (**a**) Ten clones of the Δ-δ-Ama^*Neo*^ SKO were grown in the absence of G-418 for a period of two months. The gDNA was then extracted and the presence of *neo* tested by PCR. A single band at 3.45 kb corresponding to the δ-amastin gene can be seen in the wild-type cells (WT), while an additional band at 3.65 kb corresponding to the insertion of *neo* appears in all 10 Δ-δ-Ama^*Neo*^ clones examined (*lanes 1-10*). (**b**) Cells from mid-log phase cultures of the Δ-δ-ama^*Neo*^ and Δ-γ-ama^*Hyg*^ SKOs and the Δ-γ-ama^*Hyg*^/Δ-γ-ama^*Neo*^ DKO cell lines were diluted to 1 × 10^4^ cells/ml in fresh growth medium supplemented with 500 μg/ml of the corresponding drug(s) and cells were counted every day. Values represent an average and standard deviation of three independent experiments

For establishing suitable drug resistance selection markers, first, we determined that 300 μg/ml of either G-418 or hygromycin were sufficient to kill wild-type cells following exposure for less than 48 h (Fig. 1). Next, four plasmids were constructed (pAdea γ-ama/Neo, pAdea δ-ama/Neo, pAdea γ-ama/Hyg, and pAdea δ-ama/Hyg) carrying a cassette in which the γ- or δ-amastin coding sequences (CDS) were replaced by either the neomycin (*neo*) or hygromycin (*hyg*) resistance genes that each included 1 kb each of 5’- and 3’-flanking regions (FR) from the γ- or δ-amastin gene (Additional file 2: Figure S2). After excision of the drug resistance marker gene with the genomic flanking regions (restriction sites as shown in Additional file 2: Figure S2), the excised DNA was used to transfect *A. deanei* by electroporation. Transfectants were selected in 500 μg/ml G-418 or hygromycin for approximately 2 weeks, and clonal cultures were obtained by limiting dilutions (see Methods). Analyses of cells transfected with the pAdea γ-ama/Hyg cassette revealed that HR occurred at high frequency and specifically at the targeted locus, resulting in Δ-γ-ama^*Hyg*^ single knock-out (SKO) cell lines, as indicated by (i) the occurrence of an additional PCR product at 3.6 kb using genomic DNA (gDNA) from transformants (compared to the wild-type gDNA) as a template and primers that anneal to gDNA just outside of the FR sequences used for targeting the insertion cassette (Additional file 2: Figure S3a); (ii) sequence analysis of PCR products containing the inserted cassette (Fig. 2); (iii) the occurrence of a 4.7 kbp *Apa1*/*SgrA1* gDNA band hybridizing to the hygromycin probe in Southern blot analysis (Fig. 3a); and (iv) reduced hybridization signals of the γ-amastin probe to *Apa1*/*SgrA1* restricted gDNA of the Δ-γ-ama^*Hyg*^ SKO strains relative to wild-type (Fig. 3a, compare WT with lanes 1-3). Also for the remaining 3 plasmids described in Additional file 2: Figure S2, strict HR was observed. Furthermore, integrations of the resistance cassettes were stable for over 2 months even after removing the selection pressure, as demonstrated by the PCR banding patterns of Δ-δ-ama^*Neo*^ SKO cell lines (Fig. 4a).

Since trypanosomatids are asexual diploids, the creation of null mutants requires disruption of both alleles of any specific gene. The double knock-out (DKO) Δ-γ-ama^*Hyg*^/Δ-γ-ama^*Neo*^ was readily obtained by transfection of the Δ-γ-ama^*Hyg*^ SKO cell line with the *Apa1*/*Sac1* restriction fragment from pAdea γ-ama/Neo that encodes the *neo* resistance marker flanked by γ-amastin FRs (Fig. 3b and Additional file 2: Figure S3b). In this case, restricted gDNA from the DKO strains showed no signal when the γ-amastin gene was used as a hybridization probe (Fig. 3b, panel 3, lanes 1, 2, and 3), but bands of expected sizes were observed when both *hyg* and *neo* genes were used as probes (Fig. 3b, panels 4 and 5, lanes 1, 2 and 3).

In order to test if both the δ- and γ-amastin loci can be highjacked for simultaneous expression of heterologous genes, a Δ-γ-ama^*Hyg*^/Δ-δ-ama^*Neo*^ parallel knock-out (PKO) cell line was generated (Fig. 3c and Additional file 2: Figure S3c). Insertion of the *neo* marker gene into one allele of the δ-amastin gene is shown in Fig. 3c, panels 6 and 7, while insertion of the *hyg* marker gene into one allele of the γ-amastin locus is shown in Fig. 3c, panels 8 and 9. There were no apparent phenotypical differences (e.g. cell growth) between wild-type and any of the recombinant cell lines generated, suggesting that a SKO or DKO of the γ-amastin gene, a SKO of δ-amastin, or a PKO of the δ-amastin and γ-amastin genes are not detrimental to the cells (Fig. 4b), at least under the conditions that we used for growth.

### Construction of a vector for heterologous expression of EGFP

**Fig. 5:**
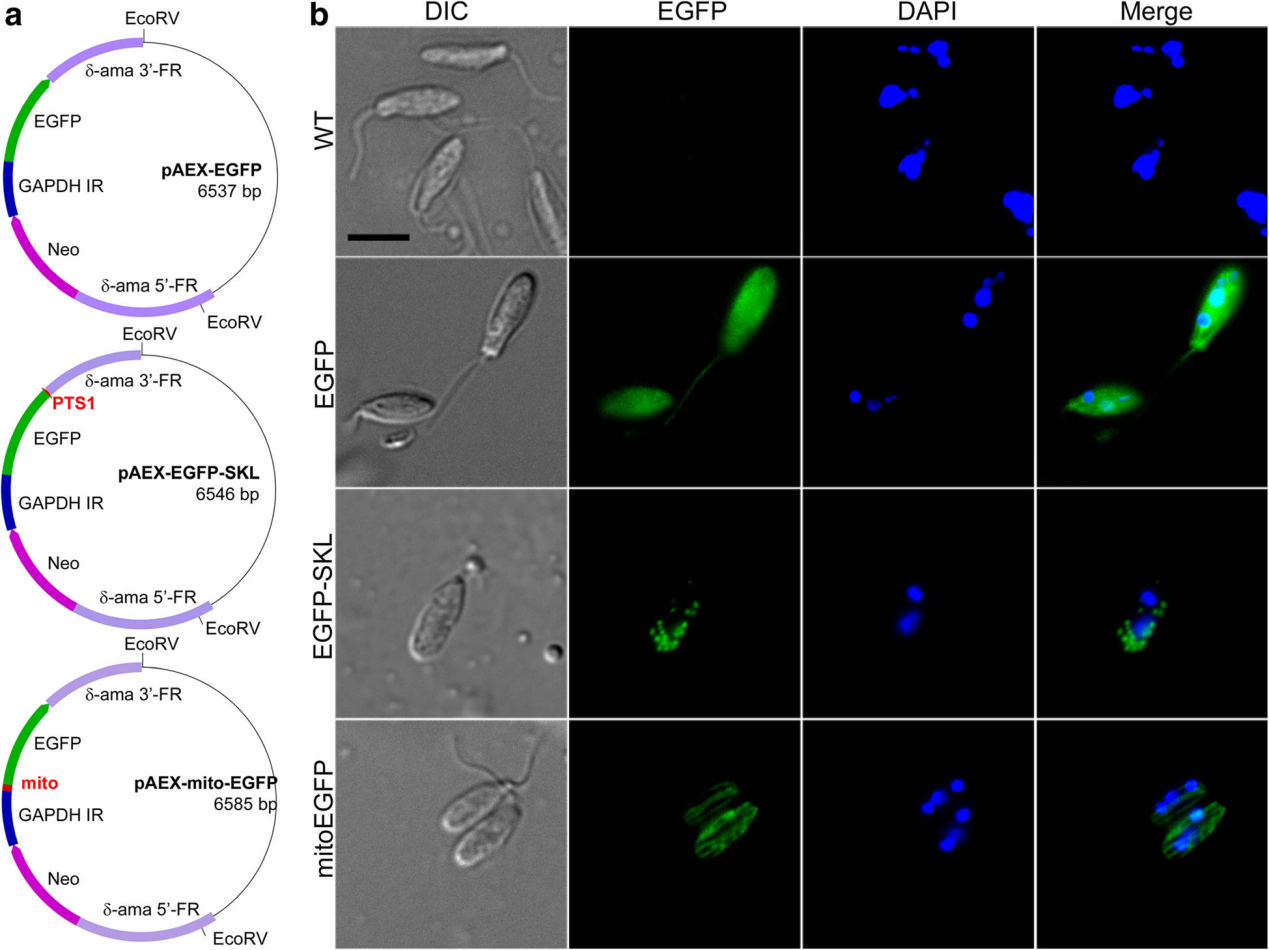
Heterologous expression and subcellular localization of EGFP in *A. deanei*. (**a**) The pAEX series of plasmids. Violet rectangles, δ-amastin 5′- and 3′-flanking regions (δ-ama FRs); blue rectangle, glyceraldehyde 3-phosphate dehydrogenase I-II intergenic region (GAPDH IR); magenta arrow, *neo*; green arrow, enhanced green fluorescence protein (EGFP); red highlights, position of the *T. brucei* dihydrolipoyl dehydrogenase mTP (mito) and the peroxisomal targeting sequence type 1 (PTS1). (**b**) Cells transfected with pAEX cassettes encoding the EGFP, mito-EGFP containing an N-terminal mTP, or EGFP-SKL containing the C-terminal PTS1 were spread on polylysine-coated glass slides, fixed in 2 % v/v ice-cold solution of paraformaldehyde in PBS, and stained with DAPI for 5 min before visualization. Scale bar: 2 μm

To facilitate protein localization studies in *A. deanei*, we used heterologous expression of the enhanced green fluorescent protein (EGFP). An expression vector (pAEX-EGFP) was constructed in which the 5’-FR of the δ-amastin gene was fused to the 5’-end of the *neo* marker gene followed by the intergenic region between the *A. deanei* glyceraldehyde 3-phosphate dehydrogenase I and II genes (GAPDH IR), a highly conserved trypanosomatid sequence containing all signals necessary for transcript maturation and stability [33, 34], and then EGFP followed by the 3’-FR of the δ-amastin gene (Fig. 5a, top). A foreign gene (encoding a protein of interest) could be integrated into this vector at the 5’ or 3’-end of the EGFP sequence, resulting in expression of an EGFP N- or C-terminal fusion protein. To achieve targeting of EGFP to various subcellular compartments, the pAEX-EGFP vector was modified by the addition of a peroxisomal targeting signal type 1 (PTS1) to EGFP, (vector pAEX-EGFP-SKL; Fig. 5a, middle), or a mitochondrial targeting peptide (mTP) from the *Trypanosoma brucei* dihydrolipoyl dehydrogenase (vector pAEX-mito-EGFP; Fig. 5a, bottom) [35]. For transfection, the expression cassettes were excised from the plasmids at the *Eco*RV restriction sites shown in Fig. 5a.

As assessed by epifluorescence microscopy, cells transfected with the pAEX-EGFP cassette showed the expected cytosolic localization of EGFP fluorescence (Fig. 5b). In contrast, cells transfected with the pAEX-EGFP-SKL cassette showed the characteristic punctate pattern of fluorescence in accord with localization of the EGFP to trypanosomatid glycosomes [36, 37], while cells transfected with the pAEX-mito-EGFP cassette showed a tubule-like fluorescence pattern that reflects the unique shape of the *A. deanei* mitochondrion [38] (Fig. 5b). These results demonstrate that (i) highjacking of the δ-amastin locus was suitable for *in vivo* expression and localization of heterologous proteins in *A. deanei,* and that (ii) using the *A. deanei* GAPDH IR to drive expression of transgenes allows for the synthesis of stable mRNAs and the subsequent accumulation of the encoded fusion protein.

### ETP1: a host-encoded protein specifically targeted to the endosymbiont

To test the hypothesis that host-encoded proteins in *A. deanei* are targeted to the endosymbiont, endosymbionts were isolated and subjected to proteomic analysis using tandem mass spectrometry. Comparative proteomic analysis of whole cell lysates versus purified endosymbionts revealed enrichment of the host-encoded ETP1 (UniProt accession S9VAC8) in the latter fraction (Table 1). Assignment of the sequence to nuclear contig number 14406 [25] and the presence of a typical SL at the 5’-end of the ETP1 mRNA clearly demonstrated its nuclear origin.

**Table 1:** The host-encoded ETP1 is enriched in the purified endosymbiont fraction. 500 ng tryptic digested protein of each sample (whole cell lysate and purified endosymbionts from three independent experiments), was analyzed by tandem mass spectrometry (see Methods)

Protein	Protein ID^*a*^	Subcellular localization^*b*^	Averaged MS/MS spectral counts^*d*^	
			Lysate	Endosymbiont
ETP1^*c*^	S9VAC8	-	12.3 ± 2.5	27.7 ± 4.0
α-Tubulin	S9WM11	Cytoskeleton	161.7 ± 8.6	7.7 ± 7.2
ATP synthase sub. α^*e*^	S9VLV1	Mitochondria	54.3 ± 7.6	5.7 ± 5.1
ATP synthase sub. β^*e*^	S9UWU8	Mitochondria	84.7 ± 2.9	11.3 ± 8.5
NADH-FRD^*f*^	S9VM53	Mitochondria & glycosomes	30.3 ± 4.2	14.0 ± 12.3

^*a*^Protein identifier corresponding to the Uniprot Database (http://www.uniprot.org)

^*b*^ *S*ubcellular localization found in other trypanosomatid species

^*c*^Endosymbiont-targeted protein 1

^*d*^Average and standard deviations were calculated from three independent experiments

^*e*^Subunit α and β of the mitochondrial ATPase complex

^*f*^NADH-dependent fumarate reductase

**Fig. 6:**
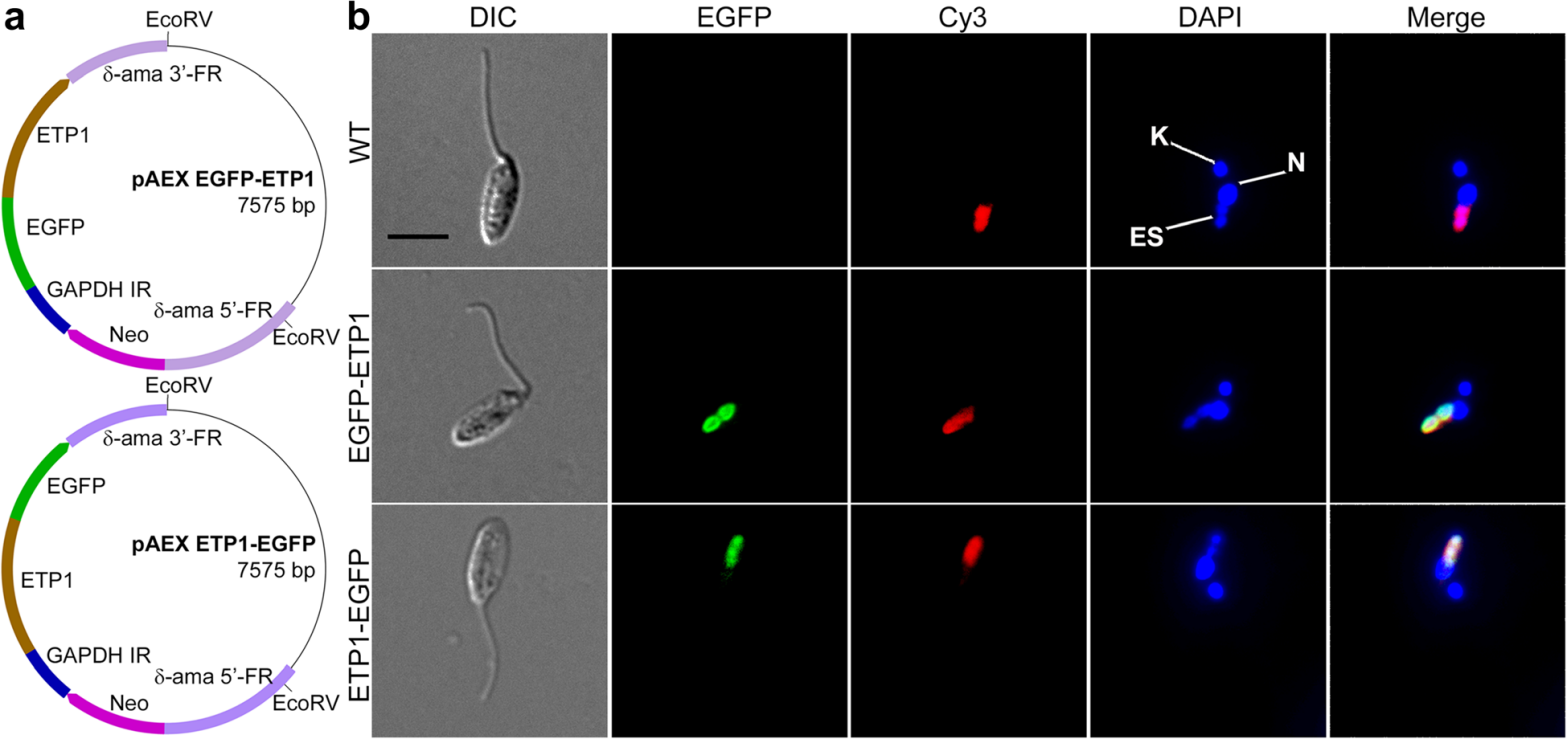
Host-encoded ETP1 is specifically targeted to the β-proteobacterial endosymbiont in *A. deanei*. (**a**) pAEX-EGFP-ETP1 and pAEX-ETP1-EGFP plasmids for expression of ETP1 fused to the C- and N-terminus of EGFP, respectively. Brown rectangle or arrow, ETP1. Remaining color code and abbreviations are as in Fig. 5. (**b**) Cells expressing the N- or C-terminal ETP1-EGFP fusion protein were analyzed by FISH-IFA using a 5′-Cy3 labeled probe against the bacterial 16S rRNA and an α-GFP IgG as described in Methods. EGFP-ETP1, N-terminal fusion of EGFP; ETP1-EGFP, C-terminal fusion of EGFP; ES, eight-shaped endosymbiont; K, kinetoplast; N, nucleus. Scale bar: 2 μm

To confirm specific targeting of ETP1 to the endosymbiont, we constructed two expression vectors encoding ETP1 fused to the N- or C-terminus of EGFP (pAEX-ETP1-EGFP and pAEX-EGFP-ETP1; Fig. 6a). *A. deanei* cells transfected with these expression cassettes were analyzed by fluorescence *in situ* hybridization (FISH) using a 5′-Cy3 labeled probe against the bacterial 16S rRNA (Eub33) [39] in combination with an immunofluorescence assay using a GFP-specific primary antibody. Epifluorescence microscopy showed specific targeting of the N- and C-terminal fusions of ETP1 to EGFP to the bacterial endosymbiont, indicating that the targeting function of the ETP1 sorting signal (which has not been characterized) was maintained in both of the fusion proteins (Fig. 6b). The same fluorescence pattern was observed by direct analysis of the EGFP signal (Additional file 2: Figure S4), however, this type of analysis cannot be combined with FISH.

## Discussion

Despite the prevalence of bacterial endosymbionts across eukaryotic phyla and their ecological and evolutionary relevance, molecular mechanisms mediating host-endosymbiont integration are largely unknown owing to a lack of effective molecular tools that enable functional studies of endosymbiotic systems. In this work, we focused on developing molecular biological tools for the trypanosomatid *A. deanei*, a promising model system for studying endosymbiosis. The ease of transfection, availability of full genome and transcriptome sequences, high frequencies of HR, and rapid growth have allowed us to generate a toolbox for efficient genetic manipulation of *A. deanei*. We established electroporation protocols, hygromycin and neomycin resistances as markers for selection of transfectants, and identified the γ- and δ-amastin loci as suitable genome sites for insertion and high level expression of transgenes. In addition, we used these newly developed tools to perform protein localization studies over a short time frame (within a month) by heterologous expression of EGFP fused to various targeting signals, with the cassette encoding these constructs integrated into the δ-amastin locus.

The ability to obtain SKO, DKO, and PKO cell lines in *A. deanei* and express fluorescent fusion proteins allows for the knock-out of specific genes, the expression of endogenous proteins with altered amino acid sequences, the establishment of over-expresser lines, the testing of heterologous proteins for activities/functions, and the localization of proteins to specific cellular compartments. These fundamental capabilities will enable, for the first time, the time and cost effective in depth dissection of the molecular mechanisms that shape the interactions between a eukaryotic host and its vertically inherited endosymbiont. Furthermore, there is the potential to expand the molecular toolbox for *A. deanei*. A recent study demonstrates that the RNAi machinery is functional in *A. deanei* and knock down of specific genes can be achieved by transfection of cells with double stranded RNAs [40]. Also the CRISPR-Cas9 system, which has recently been established in the closely related *T. cruzi* [27], can likely be applied to *A. deanei* with the tools already developed, which would help to efficiently knock out multiple genes simultaneously.

Using the newly developed tools, we firmly establish that the host-encoded protein ETP1 is specifically targeted to the endosymbiont of *A. deanei*. The absence of ETP1 orthologues in any other organism makes it difficult to predict a cellular function of this protein. Recently, it was shown that specifically inhibiting the *A. deanei* cell cycle or translation, affects the division of its endosymbiont [41]. This raises the speculative possibility that ETP1 and/or other nuclear-encoded proteins targeted to the endosymbiont might play a role in controlling its cell cycle.

Interestingly, in legume plants and insects with bacteriocyte-housed endosymbionts, proteins targeted to the bacterial endosymbionts contain an N-terminal signal peptide (SP) that routes proteins to the secretory pathway [13–15]. In contrast, *A. deanei* ETP1 is specifically targeted to its intracellular β-proteobacterium in the absence of any apparent targeting signal at its N- or C-terminus. A similar situation was reported for nuclear-encoded photosystem I proteins that are targeted to the intracellular cyanobacterium-derived organelle in the amoeba *P. chromatophora* [12]. The presence or absence of SPs in symbiont-targeted proteins probably reflects an important difference between the various endosymbiotic systems. While Rhizobia in the legumes and bacteriocyte-housed endosymbionts in insects are compartmentalized within ‘symbiosomes’ (i.e. host-derived membranes that surround the endosymbiont), the bacterial endosymbionts of *A. deanei* and *P. chromatophora* are in the host cytoplasm and not encased in a host-derived membrane. A more rigorous analysis using methods with higher resolution such as immunogold electron microscopy will be necessary to establish subcellular localization of ETP1 within the endosymbiont.

## Conclusions

The realization that symbiotic associations are ubiquitous in nature and critical to the evolution of eukaryotes makes it imperative to establish systems for elucidating the rules that govern the integration of an endosymbiotic bacterium into a eukaryotic host cell. Previous studies revealed targeting of host-encoded proteins to bacterial endosymbionts in plants (Archaeplastida), insects (Opisthokonta), and the amoeba *P. chromatophora* (Rhizaria). Here we report that also in the trypanosomatid *A. deanei* (Excavata), i.e. a member of a fourth eukaryotic superphylum, host-encoded proteins are routed to the bacterial endosymbiont. This finding suggests that the targeting of host proteins might be a general strategy of eukaryotic cells to gain control over and interact with a bacterial endosymbiont. In this work, we established *A. deanei* as a time and cost efficient reference system for in depth dissection of the molecular mechanisms that underlie host-endosymbiont interaction. Using this system to explore the partitioning of cellular functions, the trafficking of proteins and metabolites between the partner organisms and their regulatory integration, will be fundamental for our understanding of the biology of endosymbiosis and might provide insights into general principles that govern endosymbiosis as a biological phenomenon.

## Methods

### Culture conditions

*A. deanei* strain ATCC PRA-265, obtained from the American Type Culture Collection, was grown at 28 *°*C without shaking in Brain Heart Infusion (BHI, Sigma Aldrich) Broth supplemented with 10 % horse serum (Sigma Aldrich) and 10 μg/ml hemin (Sigma Aldrich). Passages of the cells were made once their density reached 1 × 10^8^ cells/ml. Cell numbers were determined by counting them in an improved Neubauer counting chamber.

### Generation of sequencing libraries, genome assembly, and RNAseq analysis

An Illumina shotgun library (insert size 400-600 bp) from *A. deanei* total gDNA was sequenced with a single Illumina MiSeq run, yielding 2 × 11,531,862 paired-end 250 bp reads. Additionally, two Nextera Mate Pair libraries from 5-6 kb and 7-10 kb gDNA fragments were sequenced with a single Illumina MiSeq run, yielding 2 × 11 million and 2 × 1.2 million paired-end 75 bp reads, respectively. The sequences were assembled *de novo* using Meraculous Assembler [42], v. 2.2.2.4 (available at https://sourceforge.net/projects/meraculous20/). The relatively modest level of polymorphism in the genome made it best suitable for assembly in diploid mode 1 which relies on identifying and traversing variant-induced "bubble" structures in the de Brujin contig graph. At an intermediate stage of the assembly process, contigs producing significant hits (Blastn) to the endosymbiont (*Candidatus* Kinetoplastibacterium crithidii TCC036E) genome, as well as PhiX contaminant contigs, were removed. Other notable assembly parameters used were k-mer size of 61 and the minimim k-mer depth cutoff of 8, the latter one aimed at removal of likely erroneous k-mers.

Trizol-extracted *A. deanei* RNA from a mid-log phase culture grown in BHI medium was used to generate a complementary DNA (cDNA) TruSeq library and sequenced (1x100 cycles) using the Illumina HiSeq protocol. The transcriptome was assembled *de novo* from 98 M quality filtered cDNA reads (phred score cutoff was 30 in at least 90 % of the bases) using the Inchworm algorithm from the Trinity software package [43] (K-mer size was 25 and minimal assembly coverage was 3). The assembly generated 16 K transcripts of >150 nt that were annotated using Blast2Go [44]. Transcript abundance levels were estimated by mapping 161 M quality filtered (phred score cutoff of 20 in at least 90 % of the bases) cDNA reads back to the assembled transcripts using Bowtie software [45] essentially as described in [46], allowing for 2 mismatches within the first 28 nucleotides and a maximal sum of mismatch Phred quality values across all the alignment of 70 (-n 2 -e 70 -l 28, --best, and --strata options). All sequencing was performed at the Stanford Functional Genomics Facility.

### Antibiotic sensitivity of *A. deanei*

Cells from mid-log phase cultures were diluted to 1 × 10^4^ cells/ml in fresh growth medium containing hygromycin B (Roth) or G-418 (Sigma Aldrich) at concentrations ranging from 50-400 μg/ml. For each concentration, cells from 3 independent cultures were counted every 12 h over a 5 day period.

### Construction of plasmids

The pAdea δ-ama/Neo, δ-ama/Hyg, γ-ama/Neo, and γ-ama/Hyg plasmids were generated by Gibson cloning using the pGEM-Teasy vector (Promega) as the backbone. Briefly, approximately 1 kb 5′- and 3′-FRs of the δ- and γ-amastin genes were amplified from *A. deanei* gDNA. The *neo* and *hyg* resistance genes were amplified from pEF1V5_HisA (Invitrogen) and pcDNA3.1/Hygro(+) (Invitrogen), respectively. The primers used to generate these DNA fragments are given in Additional file 1: Table S2. The one-pot cloning reaction using the Gibson Assembly Master Mix (New England Biolabs) contained 0.15 pmol of each purified PCR product for the 5’- and 3’-FRs, the resistance marker gene (*neo* or *hyg*), and the *Nco*I/*Pst*I-restricted pGEM-Teasy vector. Five μl of the ligation reaction was used to transform chemically competent *Escherichia coli* cells by the standard heat shock protocol (see pGEM-Teasy manual, Promega) and correct assembly of fragments was verified by analytical digestion of the vectors followed by sequencing of the replacement cassettes.

To construct the pAEX series of expression plasmids, the glyceraldehydes-3-phosphate dehydrogenase intergenic region (GAPDH IR) was amplified from *A. deanei* gDNA using the primers 030 and 031 (Additional file 1: Table S2). The resulting fragment was sub-cloned into pJET (Thermo Scientific) to generate the pJET-GAPDH IR and sequenced. Generation of the pAEX-EGFP expression plasmid was accomplished by amplifying the δ-amastin 5′-FR-*neo* and 3′-FR fragments from pAdea δ-ama/Neo, the *GAPDH* IR from pJET-GAPDH IR, and the *EGFP* gene from the vector pUMA 1445 [47]. A total of 20 fmol of each of the fragments and the pUMA 1467 backbone [47] were joined using the Golden Gate cloning system following restriction with *Bsa*I [48]. The pAEX EGFP-SKL was obtained by adding three codons coding for the PTS1 SKL immediately before the *EGFP* stop codon (see primer 037). Conversely, pAEX mito-EGFP was constructed by adding the synthetic mTP from the *T. brucei* dihydrolipoyl dehydrogenase [35] immediately after the *EGFP* start codon (see primer 040). For the construction of pAEX EGFP-ETP1 and pAEX ETP1-EGFP, the *ETP1* ORF was amplified from *A. deanei* gDNA, sub-cloned into pJET, and sequenced. An internal *Bsa*I site in the *ETP1* gene was removed by introducing a synonymous point mutation (G to A) at position 501 of the ORF. The *Bsa*I-free *ETP1* fragment was used to generate the vectors encoding the N- or C-EGFP tagged fusion proteins.

### Transfection of *A. deanei* and selection of clonal cell lines

Cells grown to mid-log phase were collected and resuspended to 1 × 10^8^ cells/ml in buffer T [25 mM 4-(2-hydroxyethyl)-1-piperazineethanesulfonic acid (HEPES), pH 7.6, 120 mM KCl, 0.15 mM CaCl_2_, 10 mM K_2_HPO_4_, 2 mM EDTA, and 5 mM MgCl_2_] or in solution for primary cells P3 (Lonza). A total of 100 μl of cells resuspended in buffer T were mixed with 10 μg of restricted cassette or full plasmid and electroporation was carried out using the program X-001 in the Nucleofector 2b (Lonza). Alternatively, 1 × 10^6^ cells were resuspended in solution P3 and mixed with 2-4 μg of restricted cassette in a final volume of 20 μl and electroporated using the program FP-158 in the Nucleofector 4D (Lonza). Following transfection, cells were transferred into 10 ml of fresh growth medium and after a 24 h recovery period, antibiotics were added to a final concentration of 500 μg/ml and were present throughout all the downstream steps unless indicated otherwise. After approximately 4 days, the transfected cells recovered and were sub-cultured in fresh medium every 3 days. At the third passage, the flagellates were diluted to a density of 1 cell/ml and aliquots of 200 μl were separated in 96-well plates. Approximately 7 days later, clonal cultures were recovered and transferred to 10 ml of fresh medium.

### Analysis of HR by PCR and Southern blot

For PCR analysis, sets of primers were designed to anneal to genomic regions upstream and downstream of the 5′- and 3′-flanking regions (FRs) of the target genes (γ- and δ-amastin), which were part of the plasmids used for transfection. Following transfection, clonal cultures of putative transformants were grown up and the gDNA of each was obtained by DNAzol treatment (Thermo Scientific) and then used as template to examine integration of transfected sequences. For Southern blots, the isolated gDNA from 5 ml culture of the selected clones was isolated with the DNAeasy Blood & Tissue kit (Qiagen) and 1 μg was restricted with the indicated enzymes, separated on 0.8 % w/v agarose gels, and transferred to nylon membranes (Nytran N Nylon Blotting Membrane, 0.45 μm; GE Healthcare Life Sciences). Labeling of the probe and DNA hybridization were performed according to the protocol supplied with the DIG-High Prime DNA Labeling and Detection Starter Kit II (Roche Applied Science) with the following hybridization temperatures: γ-amastin probe: 50°, δ-amastin probe 52 °C, and *neo*- and *hyg*-probe: 52.5 °C. Detection of the chemiluminescence on the developed membranes was performed in an ImageQuant LAS-4000 (GE Healthcare Life Sciences).

### Fluorescence microscopy

For visualization of EGFP-expressing cell lines, an aliquot of approximately 20 μl from cultures at 1 × 10^7^ cells/ml was spread on a polylysine-coated slide and incubated for 10 min. The same volume of freshly prepared ice-cold 4 % w/v paraformaldehyde (PFA) in phosphate-buffered saline (PBS) was added and after 15 min, slides were washed 3X with PBS and stained for 5 min in PBS containing 1 μg/ml DAPI. Excess dye was removed by 3 washes in PBS. For fluorescence *in situ* hybridization coupled to immunofluorescence assay (FISH-IFA), cultures were adjusted to 2 × 10^7^ cells/ml with fresh medium, mixed 1:1 with an 8 % w/v PFA solution in PBS and incubated for 30 min on ice. Aliquots of 20 μl were spotted onto agarose-coated slides, air dried and FISH was performed as described in [49, 50] using the probe Eub338 (5′-GCTGCCTCCCGTAGGAGT-3′) against the 16S bacterial rRNA coupled at its 5′-end to Cy3 [39]. After FISH, the slides were thoroughly washed with PBS and blocked with the same buffer containing 5 % v/v horse serum (HS) for 30 min followed by an additional 30 min incubation with anti-GFP rabbit IgG (Molecular Probes) at a dilution of 1:1000. After 3 washes of 5 min each with PBS + 5 % HS, slides were incubated with Alexa Fluor 488 goat anti-rabbit IgG (ThermoFisher) at a dilution of 1:250 for 30 min, washed 3X as before and stained with DAPI as described above. Images for localization studies were acquired using an epifluorescence microscope (Zeiss Axio Observer.Z1) and image manipulation and measurements were performed using the Metamorph software package (version 7, Molecular Devices).

### Purification of the endosymbiont

Endosymbionts were isolated essentially as described before [51]. All steps were performed at 4 °C and centrifugation steps for the gradients were carried out in a Beckman JS-21 using the swinging bucket rotor JS-13.1. A total of 300 ml of *A. deanei* culture grown to late-log phase was collected at 2,000 × *g* for 10 min, resuspended in 15 ml buffer A (25 mM Tris-HCl, pH 7.5, 20 mM KCl and 2 mM EDTA) containing 150 mM of sucrose and a cocktail of protease inhibitors (Roche). After sonication, the lysate was centrifuged at 7,600 × *g* for 15 min and the pellet resuspended in 8 ml of buffer A containing 250 mM of sucrose (buffer B). A total of 2 ml of the suspension was loaded on top of a discontinuous gradient of 2.5 ml 0.8 M and 5 ml of 0.4 M of sucrose in buffer A in 15 ml glass tubes (Corex) and centrifuged at 760 × *g* for 30 min. The lower whitish band obtained in the 0.4 M sucrose layer of the gradient was collected, centrifuged at 7,600 × *g* for 15 min, washed once with buffer A containing 400 mM sucrose (buffer C), resupended in 2 ml of the same buffer, and loaded on top of a percoll gradient of 2 ml each 80 %, 70 %, 60 %, 50 %, and 40 %, adjusted with the buffer A to 250 mM final concentration of sucrose; the gradient was made in 15 ml glass tubes (Corex). After centrifugation at 10,050 x *g* for 1 h, the lower three bands located between 70–60 % percoll were collected, diluted 3-5X in buffer C and centrifuged at 7,600 × g for 15 min. After removing the supernatant, the pellet was resuspended in 2 ml of buffer C and 1 ml was loaded onto an iodixanol gradient of 2.5 ml each 30 %, 27.5 %, 25 %, and 22.5 % diluted in buffer B. Following centrifugation (settings as for the previous percoll gradient), the pure endosymbiont fraction was collected from the 30-27.5 % interphase and 27.5 % iodixanol layer. This endosymbiont fraction was diluted 2X in buffer B, pelleted by centrifugation at 7,600 × g for 10 min, and the pellet resuspended in 200 μl of the same buffer and stored at -20 °C for further experiments.

### Mass spectrometric analysis of protein samples

Samples for mass spectrometry analysis were prepared as described in [52]. Peptides produced by trypsin digestion were separated on an Ultimate 3000 Rapid Separation Liquid Chromatography system (RSLC, Thermo Scientific, Dreiech, Germany) and analyzed on a Q Exactive quadrupole-orbitrap mass spectometer (Thermo Scientific, Bremen, Germany) coupled online via a nano-electrospray source. Data-dependent tandem mass spectra were acquired in positive mode and analyzed within the MaxQuant software environment (version 1.5.3.8, MPI for Biochemistry, Planegg, Germany) using standard parameters; label-free quantification was enabled and the UniProtKB proteome datasets for *A. deanei* (UP000015341) and its endosymbiont *Ca.* Kinetoplastibacterium crithidii (UP000010479), which consist of 14,609 and 739 entries, respectively, were considered for protein identification.

## Additional files

Additional file 1:
**Table S1.** mRNA abundance levels of the 120 most abundant transcripts in *A. deanei*. **Table S2.** Primers used in this study. (PDF 169 kb)
Additional file 2:
**Figure S1.** Sequences of the targeted γ- and δ-amastin ORFs. **Figure S2.** pAdea series of plasmids containing the neomycin and hygromycin replacement cassettes. **Figure S3.** High frequency of homologous recombination in *A. deanei*. **Figure S4.** EGFP fluorescence pattern suggests targeting of host-encoded ETP1 to the endosymbiont in *A. deanei*. (PDF 736 kb)
