# Supplementary material for: Development of a toolbox to dissect host-endosymbiont interactions and protein trafficking in the trypanosomatid Angomonas deanei
Source: BMC Evol Biol. 2016 Nov 11;16:247. doi: 10.1186/s12862-016-0820-z (PMC5106770; doi:10.1186/s12862-016-0820-z)
Supplement: Additional file 1: — Table S1. mRNA abundance levels of the 120 most abundant transcripts in A. deanei. Table S2. Primers used in this study. (PDF 169 kb) [file 12862_2016_820_MOESM1_ESM.pdf]

Supplementary Tables

Table S1: mRNA abundance levels of the 120 most abundant transcripts in *A. deanei*. The table provides the name of the *de novo* assembled transcript, length of the transcript, number of reads mapping to each transcript, transcript abundance (reads per kbp), and annotation. δ- and γ-amastin are highlighted in bold.

Rank	Transcript Name	Length [nt]	No. of Reads	Reads/ kbp	Annotation
1	a4;46817	1547	948701	613,252	elongation factor 1-alpha
2	a3;44156	1642	911325	555,009	alpha tubulin
3	a5;32850	1566	655183	418,380	beta tubulin
4	a2;48346	331	124332	375,625	---NA---
5	a10;28593	1291	482986	374,118	glycosomal GAP-DH
6	a6;26072	2567	858524	334,446	surface antigen-like protein
7	a18;24067	1598	514389	321,895	enolase
8	a12;23665	2496	789514	316,312	heat-shock protein hsp70
9	a11;22235	2041	596168	292,096	indolepyruvate decarboxylase
10	a7;22144	714	203364	284,824	cytochrome c
11	a13;26409	857	243522	284,156	flagellar calcium-binding protein
12	a23;22353	509	142119	279,212	60s ribosomal protein l44
13	a1;42697	304	84491	277,931	beta tubulin
14	a22;18919	1353	340364	251,562	fructose-bisphosphate class i
15	a27;19289	716	173417	242,203	60s ribosomal protein l9
16	a20;18396	552	119820	217,065	calpain-like cysteine peptidase
17	a31;21873	276	59858	216,877	polyubiquitin
18	a14;17757	756	160680	212,540	---NA---
19	a26;16762	941	199867	212,399	40s ribosomal protein sa
20	a9;24571	226	46144	204,177	---NA---
21	a41;15167	1994	400811	201,009	glycosomal PEP carboxykinase
22	a19;15795	870	172857	198,686	tryparedoxin peroxidase
23	a51;14954	2366	467148	197,442	heat shock protein 83-1
24	a43;15671	761	149228	196,095	60s ribosomal protein l10a
25	a36;16096	564	108226	191,890	40s ribosomal protein s23
26	a34;14924	1758	336895	191,635	alcohol dehydrogenase
27	a8;17332	2477	472841	190,893	beta-fructofuranosidase-like protein
28	a24;18393	420	79908	190,257	---NA---
29	a38;14473	550	99165	180,300	40s ribosomal protein s24e
30	a30;13553	456	81964	179,746	calpain-like cysteine peptidase
31	a58;12880	1054	184211	174,773	60s ribosomal protein l5
32	a48;13243	1117	191004	170,997	carrier protein mitochondrial precursor
33	a61;13029	908	152020	167,423	40s ribosomal protein s3a
34	a74;12633	1290	206669	160,209	activated protein kinase c partial
**35**	**a96;12664**	**760**	**120574**	**158,650**	**δ-amastin**
36	a73;11995	856	135615	158,429	60s ribosomal protein l7
37	a50;12518	738	116068	157,274	40s ribosomal protein s9
38	a56;12698	631	98538	156,162	40s ribosomal protein s11
39	a15;12082	7507	1169704	155,815	senescence-associated protein
40	a33;12453	626	97176	155,233	40s ribosomal protein s21
41	a88;11976	811	124828	153,919	40s ribosomal protein s3
42	a85;11921	526	80410	152,871	60s ribosomal protein l27a l29
43	a60;12230	607	92255	151,985	40s ribosomal protein s13
44	a86;11974	610	90648	148,603	60s ribosomal protein l17
45	a21;14775	351	52099	148,430	---NA---
46	a59;11781	1404	207634	147,887	ribosomal protein l3
47	a29;11531	880	127359	144,726	40s ribosomal protein s6
48	a78;11225	1107	157172	141,980	60s acidic ribosomal subunit protein
49	a97;10553	895	124674	139,301	40s ribosomal protein s2
50	a45;12605	463	64095	138,434	kinetoplastid membrane protein-11
51	a68;10393	544	74932	137,743	40s ribosomal protein s17
52	a70;10886	566	76913	135,889	60s ribosomal protein l22
53	a46;11445	504	68472	135,857	40s ribosomal protein s16
54	a25;11046	1005	136001	135,324	hypothetical protein AGDE_14034
55	a77;10694	658	88866	135,055	60s ribosomal subunit protein l31
56	a100;10208	1899	252084	132,746	glutamate dehydrogenase

57	a71;10389	803	106270	132,341	60s ribosomal protein l19
58	a106;9562	607	78778	129,783	60s ribosomal protein l12
59	a103;9859	833	107713	129,307	iron superoxide dismutase
60	a82;9768	578	74244	128,450	60s ribosomal protein l21
61	a76;10098	717	92052	128,385	40s ribosomal protein s12
62	a99;10138	630	80087	127,122	40s ribosomal protein l14
63	a91;10827	729	92555	126,962	40s ribosomal protein s19 protein
64	a120;9779	796	100502	126,259	60s ribosomal protein l10
65	a107;10205	774	97542	126,023	60s ribosomal protein l13
66	a110;9860	640	79871	124,798	60s ribosomal protein l18a
67	a52;9335	671	83629	124,633	60s ribosomal protein l23
68	a81;10201	514	63621	123,776	ribosomal protein s20
69	a90;9522	743	91115	122,631	ribosomal protein l15
70	a80;9375	539	65901	122,265	60s ribosomal protein l26
71	a111;9243	1268	154793	122,076	s-adenosylmethionine synthetase
72	a54;9656	536	65057	121,375	40s ribosomal protein s15a
73	a35;10167	532	64474	121,192	40s ribosomal protein s18
74	a65;11056	310	37372	120,555	40s ribosomal protein s30
75	a92;8800	2753	331216	120,311	elongation factor 2
76	a32;18792	150	17792	118,613	---NA---
77	a112;8825	729	85670	117,517	ribosomal protein s7
78	a53;14321	209	24522	117,330	---NA---
79	a134;8988	804	93307	116,053	60s ribosomal protein l13a
80	a102;9176	943	109408	116,021	40s ribosomal protein s4
81	a94;9445	563	65157	115,732	60s ribosomal protein l23a
82	a95;8918	986	112857	114,459	60s ribosomal protein l2
83	a55;8910	642	73356	114,262	ribosomal protein s25
84	a63;9693	800	90198	112,748	40s ribosomal protein s5
85	a127;8752	1410	158320	112,284	heat shock 70-related protein precursor
86	a75;9254	574	63058	109,857	ubiquitin ribosomal protein s27a
87	a122;8745	1210	132911	109,844	glycosomal malate dehydrogenase
88	a69;8906	2212	241438	109,149	hypothetical protein, conserved (fragment)
89	a17;10008	993	106630	107,382	---NA---
90	a67;8726	619	66070	106,737	40s ribosomal protein s10
91	a64;8769	580	61645	106,284	60s ribosomal protein l30
92	a44;8400	855	90115	105,398	60s ribosomal protein l18
93	a124;8295	773	80999	104,785	40s ribosomal protein s8
94	a101;7898	542	56782	104,764	60s ribosomal protein l35
95	a47;10045	484	50303	103,932	atpase subunit 9
96	a42;8729	381	39473	103,604	60s ribosomal protein l37a
97	a132;8248	729	75098	103,015	glycine cleavage system h protein
98	a114;8032	832	85659	102,956	eukaryotic initiation factor 5a
99	a140;8347	767	77091	100,510	60s ribosomal protein l6
100	a98;7686	552	54985	99,611	60s ribosomal protein l36
101	**a66;8439**	**1028**	**101711**	**98,941**	**γ-amastin**
102	a93;8119	601	59098	98,333	60s ribosomal protein l28
103	a158;9402	352	34281	97,389	beta-fructofuranosidase-like protein
104	a139;7175	1686	158104	93,775	myo-inositol-1-phosphate synthase
105	a57;8357	338	31639	93,607	ribosomal protein l29
106	a28;7176	607	56744	93,483	histone h2b
107	a89;7902	364	32817	90,157	ribosomal protein l38
108	a83;7828	635	56822	89,483	60s ribosomal protein l32
109	a105;8324	350	31312	89,463	ribosomal protein s29
110	a128;7001	991	88410	89,213	60s ribosomal protein l7a
111	a130;6549	812	69855	86,028	ribonucleoprotein mitochondrial precursor
112	a123;6680	568	48499	85,386	40s ribosomal protein s15
113	a142;7283	446	37997	85,195	---NA---
114	a118;7068	634	53697	84,696	nascent polypeptide associated complex subunit-like copy 1
115	a104;6297	660	55813	84,565	40s ribosomal protein s14
116	a117;6934	1324	111922	84,533	---NA---
117	a149;6877	898	75535	84,115	heat shock 70-related protein precursor
118	a16;8469	907	76050	83,848	histone h4
119	a135;6719	474	39586	83,515	ribosomal protein l24
120	a175;6389	500	41611	83,222	cofilin-like protein

Table S2: Primers used in this study.

Name	Internal Name	Purpose	Sequence	
001	5'flAma_fw (pGEM_5'flank_fw)	amplification of δ-ama 5'-FR with pGEM-Teasy overhang	atatggtcgacctgcaCAGTGCGTCGCCCGGCTA	**Construction of pAdea δ-ama/Hyg**
002	5'flAma_rev (5'flank_Hyg_rev)	amplification of δ-ama 5'-FR with *hyg* overhang	ggctttttcatTTTCTTGTGAGATGATTATAAAAAGTGTTTTTTTTAAAAAAAAAAAG	
003	Hyg_fw (5'flank_Hyg_fw)	amplification of *hyg* with δ-ama 5'-FR overhang	cacaagaaaATGAAAAAGCCTGAACTCACC	
004	Hyg_rev (Hyg_3'flank_rev)	amplification of *hyg* with δ-ama 3'-FR overhang	ccctcttaTTTCTTTGCCCTCGGACG	
005	Hyg_3'flank_fw	amplification of δ-ama 3'-FR with *hyg* overhang	gcaaagaaaTAAGAGGGGGGAGAGAGAC	
006	3'flank_pGEM_rw	amplification of δ-ama 3'-FR with pGEM-Teasy overhang	catgctcccggccgcCCTTTTTTTTACTGGATCG	
007	5'Fl_fw	amplification of δ-ama 5'-FR with pGEM-Teasy overhang	agctctcccatatggtcgacctgcaCAGTGCGTCGCCCGGCTA	**Construction of pAdea δ-ama/Neo**
008	5'Fl rev (Neo)	amplification of δ-ama 5'-FR with *neo* overhang	cgtgcaatccatcttgttcaatcatTTTCTTGTGAGATGATTATAAAAAGTGTTTTTTTTAA	
009	Neo_fw (Ama)	amplification of *neo* with δ-ama 5'-FR overhang	aaacacttttataatcatctcacaagaaaATGATTGAACAAGATGGATTGCAC	
010	BS_Neo_rev (Ama)	amplification of *neo* with δ-ama 3'-FR overhang	actcacgtctctctccccctcTTAGAAGAACTCGTCAAGAAGGC	
011	3'Fl_fw (Neo)	amplification of δ-ama 3'-FR with *neo* overhang	tcgccttcttgacgagttcttctaaGAGGGGGGAGAGAGACGT	
012	3'Fl rev	amplification of δ-ama 3'-FR with pGEM-Teasy overhang	cgacgtcgcatgctcccggccgCCTTTTTTTTACTGGATCGCGCG	
013	5'G_fw	amplification of γ-ama 5'-FR with pGEM-Teasy overhang	tcccatatggtcgacctgcaGATGATTTGCAAGTGCATG	**Construction of pAdea γ-ama/Hyg**
014	5'G_rev (Hyg)	amplification of γ-ama 5'-FR with *hyg* overhang	gtgagttcaggctttttcatTCTTTCTTTTTTTTTATTTTATTTTATTTTTGATGG	
015	Hyg_fw(G)	amplification of *hyg* with γ-ama 5'-FR overhang	ccatcaaaaataaaataaaataaaaaaaaagaaagaATGAAAAAGCCTGAACTCACCG	
016	Hyg_rev(G)	amplification of *hyg* with γ-ama 3'-FR overhang	ctgttttttgttttgttttgtatgtttttaTTTCTTTGCCCTCGGACGA	
017	3'G_fw(Hyg)	amplification of γ-ama 3'-FR with *hyg* overhang	cagcactcgtccgagggcaaagaaaTAAAAAACATACAAAACAAAACAAAAAAACAG	
018	3'G_rev	amplification of γ-ama 3'-FR with pGEM-Teasy overhang	cgtcgcatgctcccggccgcAGTGATTCAATAGCGGTAATAAAC	
013	5'G_fw	amplification of γ-ama 5'-FR with pGEM-Teasy overhang	tcccatatggtcgacctgcaGATGATTTGCAAGTGCATG	**Construction of pAdea γ-ama/Neo**
019	gAma_5'fl_rev (Neo)	amplification of γ-ama 5'-FR with *neo* overhang	cgtgcaatccatcttgttcaatcatTCTTTCTTTTTTTTTATTTTATTTTATTTTTGATGG	
020	Neo_fw(G)	amplification of *neo* with γ-ama 5'-FR overhang	ccatcaaaaataaaataaaataaaaaaaaagaaagaATGATTGAACAAGATGGATTGCAC	
021	Neo_rev(G)	amplification of *neo* with γ-ama 3'-FR overhang	ctgttttttgttttgttttgtatgtttttaGAAGAACTCGTCAAGAAGGC	
022	gAma_3'fl_fw(Neo)	amplification of γ-ama 3'-FR with *neo* overhang	ctatcgccttcttgacgagttcttcTAAAAAACATACAAAACAAAACAAAAAAACAG	
023	3'G_rev	amplification of γ-ama 3'-FR with pGEM-Teasy overhang	cgtcgcatgctcccggccgcAGTGATTCAATAGCGGTAATAAAC	
024	5'neonostopBsaI FW	Amplification of δ-ama5'-neo fragment	GGTCTCGCCTGCAGTGCGTCGCCCGGCTA	**Construction of pAdea Neo-GFP fusion**
025	5'neonostopBsaI RV	Amplification of δ-ama 5'-neo fragment	GGTCTCGACGAAGAACTCGTCAAGAAGGCGATAGAA	
026	GFP-3'bsaI FW	Amplification of δ-ama 3'	GGTCTCGAAAGAGGGGGGAGAGAGAC	
027	3-pUMAmaBsaI RV	Amplification of δ-ama 3'	GGTCTCGCTGCGTCGCGGGGGCTGTCGCA	
028	GFPnostartBsaI FW	Amplification of EGFP	GGTCTCCTCGTGAGCAAGGGCGAGGAG	
029	GFPGGBsaI RV	Amplification of EGFP	GGTCTCCCTTTTACTTGTACAGCTCGTCCATGC	
030	GAPDHIR FW	Amplification GAPDH IR	AAGTGCTAGTGAGAGTTTGACT	
031	GAPDPHIR RW	Amplification GAPDH IR	TTGGATAACTGTGTTTTTTGATG	

024	5'neonostopBsaI FW	Amplification of δ-ama5′-neo fragment	GGTCTCGCCTGCAGTGCGTCGCCCGGCTA	**Construction of pAEXEGFP**
032	δ-Ama5'-GAPDH RV	Amplification of δ-ama5′-neo fragment	GGTCTCGACTTTTAGAAGAACTCGTCAAGAAGGC	
033	GAPDH-δAma 3' FWD	Amplification of EGFP-δ-ama 3′fragment	GGTCTCGCAAATGGTGAGCAAGGGCGAGGAGC	
027	3-pUMAmaBsaI RV	Amplification of EGFP-δ-ama 3′ fragment	GGT CTCGCTGCGTCGCGGGGGCTGTCGCA	
034	δ-Ama5'-GAPDH FW	Amplification GAPDH IR	GGTCTCGAAGTGCTAGTGAGAGTTTGACTCG	
035	GAPDH-δAma 3' RV	Amplification GAPDH IR	GGTCTCGGTTGGATAACTGTGTTTTTTGATGAAAGAAG	
024	5'neonostopBsaI FW	Amplification of δ-ama5′-neo-GAPDH IR fragment	GGTCTCGCCTGCAGTGCGTCGCCCGGCTA	**Construction of pAEX EGFP-SKL**
035	GAPDH-δAma 3' RV	Amplification of δ-ama5′-neo-GAPDH IR fragment	GGTCTCGGTTGGATAACTGTGTTTTTTGATGAAAGAAG	
036	GFP-SKL FW	Amplification of EGFP containing PTS1	CTCGGTCTCCCCAAATGGTGAGCAAGGGCGAG	
037	GFP-SKL RV	Amplification of EGFP containing PTS1	CTCGGTCTCCCTTTTACAGTTTGGACTTGTACAGCTCG	
038	GFPSKL-δ-ama3' FW	Amplification of EGFP-δ-ama 3′ fragment	CTCGGTCTCCAAAGAGGGGGGAGAGAGACG	
027	3-pUMAmaBsaI RV	Amplification of EGFP-δ-ama 3′ fragment	GGTCTCGCTGCGTCGCGGGGGCTGTCGCA	
024	5'neonostopBsaI FW	Amplification of δ-ama5′-neo-GAPDH IR fragment	GGTCTCGCCTGCAGTGCGTCGCCCGGCTA	**Construction of pAEX mitoEGFP**
039	GAPDH-mitoGFP RV	Amplification of δ-ama5′-neo-GAPDH IR fragment	AGCGGTCTCGTTGGATAACTGTGTTTTTTGATGAAAGAAGAC	
040	mitoGFP FW	Amplification of mitoEGFP-δ-ama 3′ fragment	AGCGGTCTCGCCAAATGAAGCTTATGTTTCGTCGTTGCTTTCCCATT TTTAACCCCTACGACGTGGTGAGCAAGGGCGAGGAGCT	
027	3-pUMAmaBsaI RV	Amplification of mitoEGFP-δ-ama 3′ fragment	GGTCTCGCTGCGTCGCGGGGGCTGTCGCA	
041	Ama5'UTRsc2 FW	Sequencing insertion in δ-amastin 5′	ACTCCTCCACCACTACCACC	
042	Neo internal RV	Sequencing insertion in δ-amastin 5′	ATCGACAAGACCGGCTTCC	
043	Neo internal FW	Sequencing insertion in δ-amastin 3′	CGAAACATCGCATCGAGCG	
044	Ama3'UTRsc RV	Sequencing insertion in δ-amastin 3′	AGAGAGAGGTGGGTACATGCAA	
045	gama-Ama-region FW	Sequencing insertion in γ-amastin 5′	CTTTCTGCCATCTGCCTCAT	
046	Hyg internal RV	Sequencing insertion in γ-amastin 5′	AAAGCATCAGCTCATCGAGAG	
047	Hyg internal FW	Sequencing insertion in γ-amastin 3′	TGTGTATCACTGGCAAACTGTG	
048	gama-Ama-region RV	Sequencing insertion in γ-amastin 3′	CATCCTTACGATCTTCTTATTTTTGG	
024	5'neonostopBsaI FW	Amplification of δ-ama5′-neo-GAPDH IR fragment	GGTCTCGCCTGCAGTGCGTCGCCCGGCTA	**Construction of pAEX Etp1-EGFP**
049	pAEXCand1-c REV(2)	Amplification of δ-ama5′-neo-GAPDH IR fragment	GGTCTCCTTTGGATAACTGTGTTTTTTGATGAAAGAAGAC	
050	pAEXCand1-c FWD (3)	Amplification of Etp1 gene	GGTCTCCCAAATGCCCGCCGCGCTGCCC	
051	pAEX Cand1-cREV(3)	Amplification of Etp1 gene	GGTCTCCCACCGCATCCTTCTTCTCTACA	
052	pAEX Cand1-c FWD(4)	Amplification of EGFP-δ-ama 3′ fragment	GGTCTCCGGTGAGCAAGGGCGAGGAG	
027	3-pUMAmaBsaI RV	Amplification of EGFP-δ-ama 3′ fragment	GGTCTCGCTGCGTCGCGGGGGCTGTCGCA	

024	5'neonostopBsaI FW	Amplification of δ-ama5'-neo-GAPDH IR-EGFP fragment	GGTCTCGCCTGCAGTGCGTCGCCCGGCTA	**Construction of pAEX EGFP-Etp1**
053	pAEXCand1-nGFP REV (2)	Amplification of δ-ama5'-neo-GAPDH IR-EGFP fragment	GGTCTCCGGCTTGTACAGCTCGTCCAT	
054	pAEXCand1-nGFP FWD (3)	Amplification of Etp1 gene	GGTCTCGAGCCCGCCGCGCTGCCCCCC	
055	pAEXCand1-n REV (3)	Amplification of Etp1 gene	GGTCTCGCTTTTACGCATCCTTCTTCTCTACAACATCCGA	
056	pAEXCand1-n FWD (4)	Amplification of δ-ama 3'	GGTCTCCAAAGAGGGGGGAGAGAGAC	
027	3-pUMAmaBsaI RV	Amplification of δ-ama 3'	GGTCTCGCTGCGTCGCGGGGGCTGTCGCA	
057	d-Amastin probe fw	Generation of δ-amastin probe	ACTACTGCGTGACCCTCTGG	**Generation of Southern blot probes**
058	d-Amastin probe rv	Generation of δ-amastin probe	AGTCGTCGTGCTTGTTGTTG	
059	g-Amastin probe fw	Generation of γ-amastin probe	CCGCTTTTGAGATTGTCTCC	
060	g-ama rev	Generation of γ-amastin probe	GGCGGCGTAAATGTTAAAGA	
061	hygro probe fw	Generation of hyg probe	ATCTTAGCCAGACGAGCG	
062	hygro probe rev	Generation of hyg probe	CACTATCGGCGAGTACTTCTACA	
063	neo probe fw	Generation of neo probe	TTGTCAAGACCGACCTGTC	
064	neo probe rev	Generation of neo probe	CAAGAAGGCGATAGAAGGC	
