# Supplementary material for: Development of a toolbox to dissect host-endosymbiont interactions and protein trafficking in the trypanosomatid Angomonas deanei
Source: BMC Evol Biol. 2016 Nov 11;16:247. doi: 10.1186/s12862-016-0820-z (PMC5106770; doi:10.1186/s12862-016-0820-z)
Supplement: Additional file 2: — Figure S1. Sequences of the targeted γ- and δ-amastin ORFs. Figure S2. pAdea series of plasmids containing the neomycin and hygromycin replacement cassettes. Figure S3. High frequency of homologous recombination in A. deanei. Figure S4. EGFP fluorescence pattern suggests targeting of host-encoded ETP1 to the endosymbiont in A. deanei. (PDF 736 kb) [file 12862_2016_820_MOESM2_ESM.pdf]

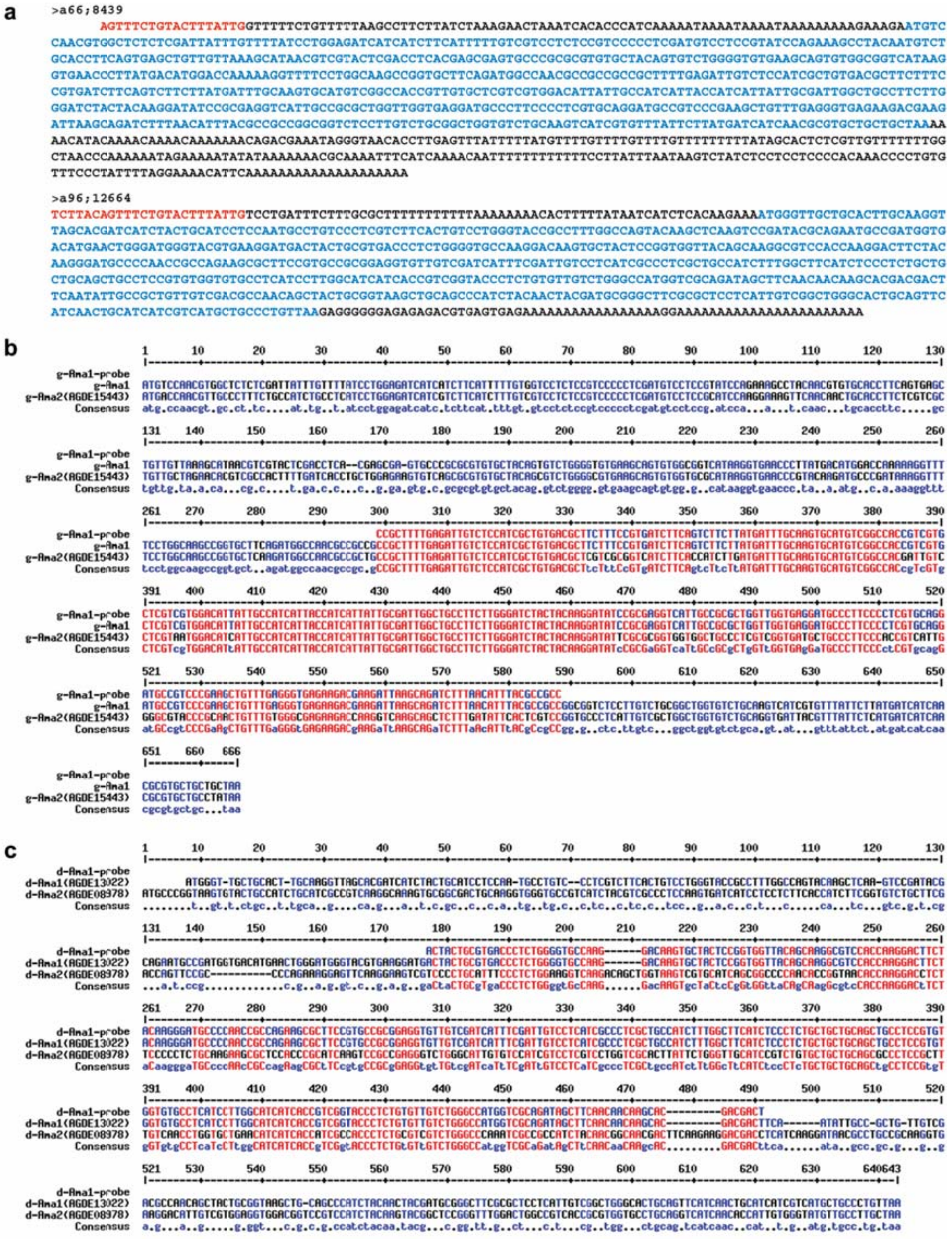

Figure S1. Sequences of the targeted γ- and δ- amastin ORFs. **(a)** Transcripts for the targeted γ-amastin (A66; 8469) and δ-amastin (a96; 12664) family genes showing the splice leader (red) and ORF (blue), respectively. **(b, c)** Alignments of the targeted γ-amastin and δ-amastin (AGDE13022) with their closest variants (AGDE15443 for γ-amastin and AGDE08978 for δ-amastin) as well as the probe used for the Southern blots. Note that both targeted genes are sufficiently dissimilar from to their closest variant to yield a specific signal in Southern blot analyses using the protocol described.

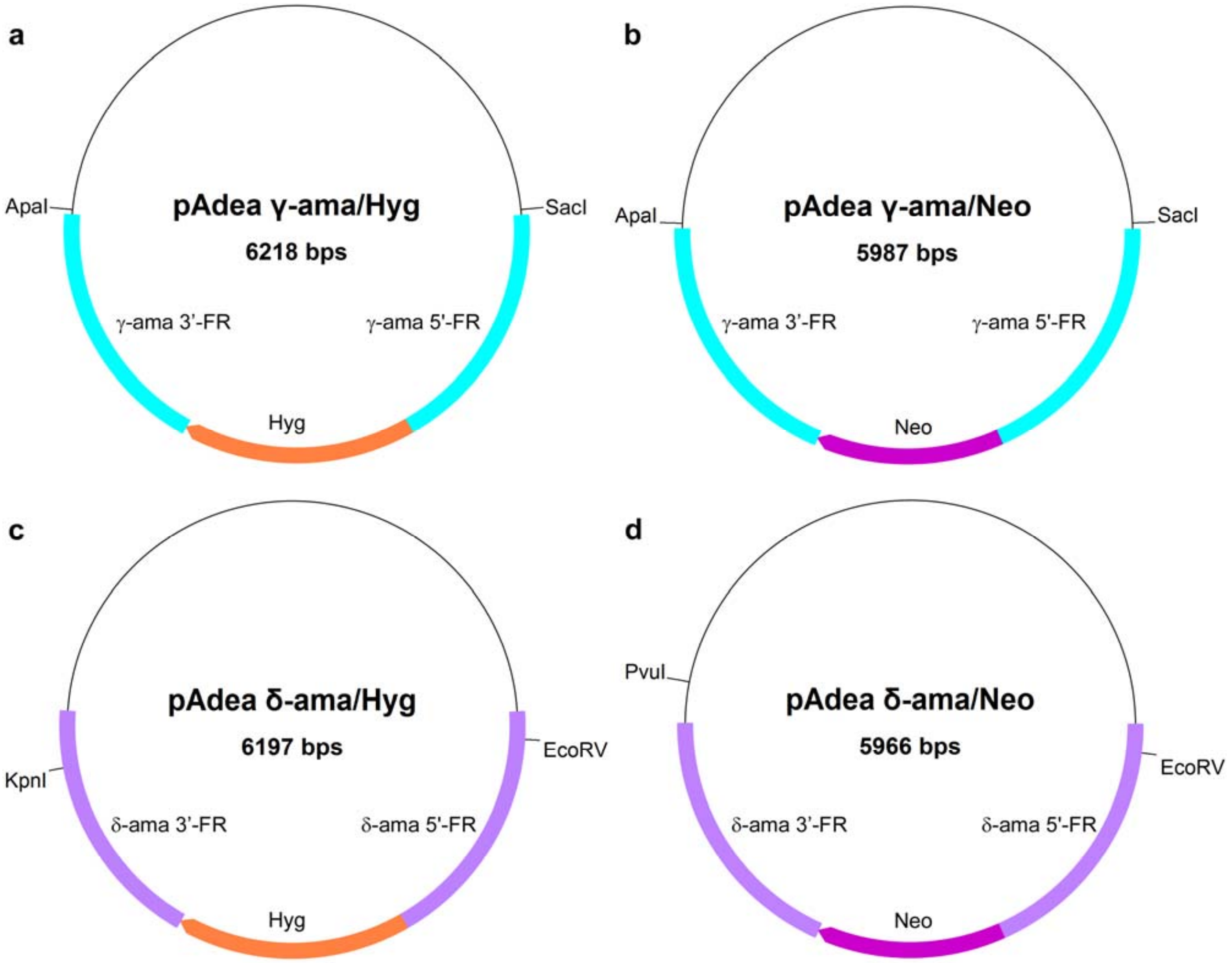

Figure S2: pAdea series of plasmids containing the neomycin and hygromycin replacement cassettes. Fragments of 1 kb of the 5'- and 3'-FRs of the γ-amastin gene (**a** and **b**) or δ-amastin gene (**c** and **d**) were amplified from *A. deanei* gDNA and the selectable marker genes (*hyg* or *neo*) were inserted between the FRs; the entire γ- or δ-amastin CDS was eliminated and replaced with the drug resistant gene maintained in the same orientation as the original amastin sequence. The restriction sites indicate the excision product used for transfections described in this work. Vector backbone: pGEM-Teasy.

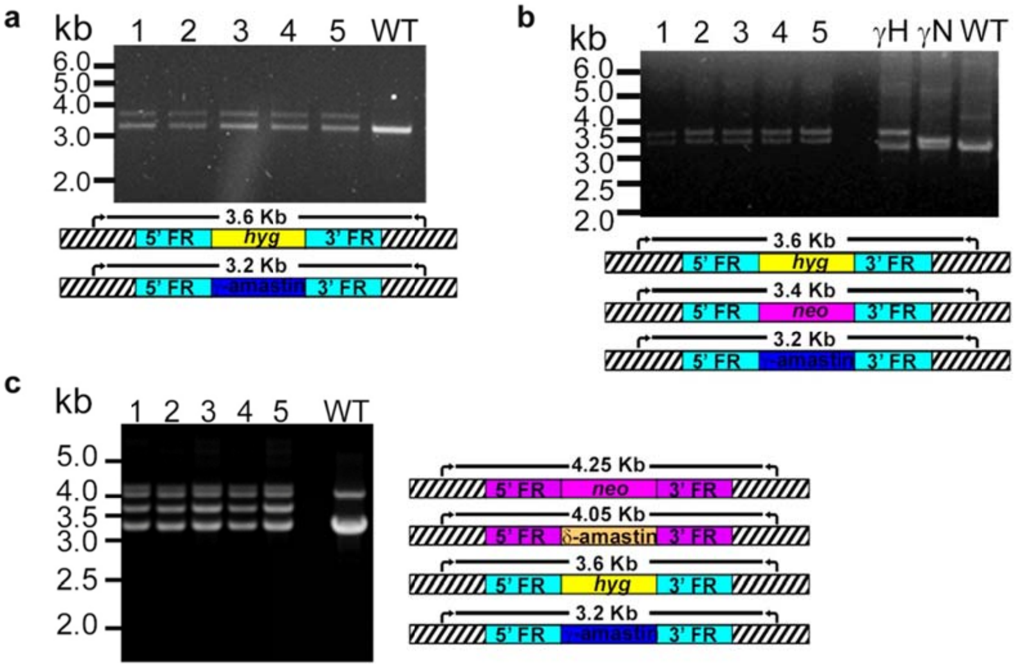

Figure S3: High frequency of homologous recombination in *A. deanei*. Around 2 weeks after transfection, clonal cultures were generated by limiting dilution. A week later, ~20 clones were picked, the gDNA extracted, and the insertion analyzed by PCR. Results are shown for 5 clones each, but all clones picked yielded the same banding patterns. **(a)** A single band at 3.2 kb corresponding to the γ-amastin locus can be seen in wild-type cells (WT) while an additional band at 3.6 kb corresponding to the insertion of *hyg* appears in all of the Δ-γ-ama^{Hyg} SKO clones examined; **(b)** in all Δ-γ-ama^{Hyg}/Δ-γ-ama^{Neo} DKO clones examined, the expected bands for the insertion of *hyg* and *neo* appear at 3.6 and 3.4 kb, respectively, while the band at 3.2 kb that corresponds to the WT γ-amastin locus disappears; and **(c)** in addition to the bands observed in (a), two PCR products at 4.05 and 4.25 kb corresponding to the WT δ-amastin and *neo* recombinant loci were detected in all Δ-γ-ama^{Hyg}/Δ-δ-ama^{Neo} PKO cell lines tested. γH corresponds to the Δ-γ-ama^{Hyg} and γN to the Δ-γ-ama^{Neo} SKO cell lines used as controls.

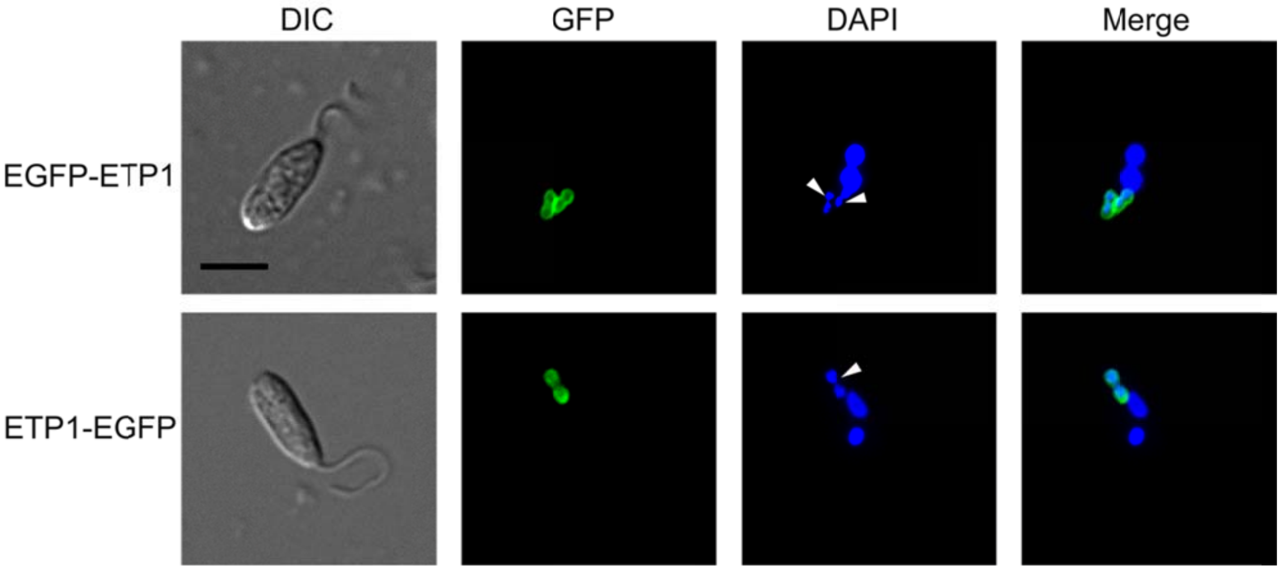

Figure S4: EGFP fluorescence pattern suggests targeting of host-encoded ETP1 to the endosymbiont in *A. deanei*. Cells expressing the N- or C-terminal ETP1-EGFP fusion protein were fixed in PFA, DAPI stained, and analyzed by epifluorescence microscopy. EGFP-ETP1, N-terminal fusion of EGFP; ETP1-EGFP, C-terminal fusion of EGFP; white arrow heads highlight the eight-shaped endosymbiont in the DAPI panel. Scale bar: 2 µm.
